# 2672. Understanding the Barriers to Sexual Health in Middle Eastern Immigrant Women

**DOI:** 10.1093/ofid/ofad500.2283

**Published:** 2023-11-27

**Authors:** Noora Kazanji, Hana Masri, Laura Power

**Affiliations:** Corewell Health, Bloomfield Hills, Michigan; Arab Community Center for Economic and Social Services, Dearborn, Michigan; University of Michigan School of Public Health, Ann Arbor, Michigan

## Abstract

**Background:**

Sexually transmitted infections (STI) are often considered taboo in the life of women living in the Middle East and North Africa (MENA), and few studies have evaluated how to raise awareness of sexual health (SH) for these women. There are no studies that have aimed to understand if social norms, stigmas, and misconceptions surrounding seeking SH care continue for women who have immigrated to the United States (US) from MENA countries. The primary goal of this study is to determine existing cultural barriers to seeking SH care among US-living women who have emigrated from MENA or whose parents have emigrated from MENA.

**Methods:**

From April 2021 to August 2021, 18 semi-structured interviews were conducted in women who emigrated from MENA or whose parents emigrated from MENA. We used thematic content analysis to identify the main themes and subthemes around barriers to seeking SH care. We then developed a conceptual model to depict how different themes interact to influence attitudes and beliefs towards SH care of MENA immigrant women.

**Figure:**
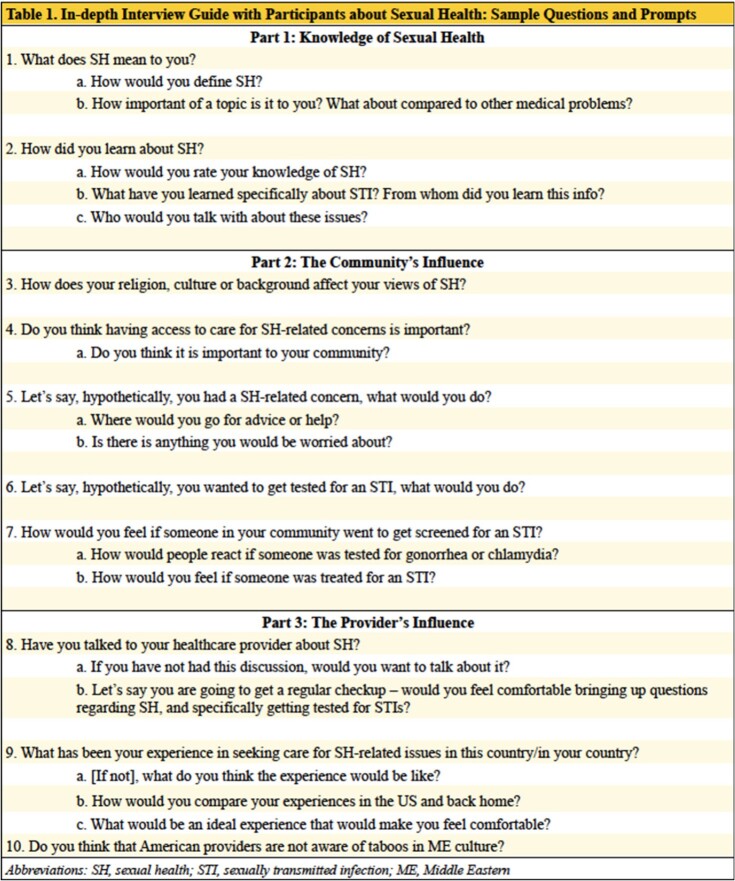

**Results:**

The primary themes that emerged were (a) *reduced knowledge about SH resources and STI*, (b) *judgement*, (c) *lack of discussion in the community and household*, (d) *circumvention*, and (e) *call to action*. The subtheme of cultural expectation of abstinence before marriage was a cross-cutting theme brought up by 16 of the 18 participants. One of the subthemes within the theme of *judgement* was the preference to seek SH care outside the community. Participants felt there was a barrier with discussing SH concerns with physicians who are of the same community due to the perception of judgement from that physician with whom the participant identifies. *Call to action* highlights several solutions shared by participants to reduce barriers to SH care.

**Figure:**
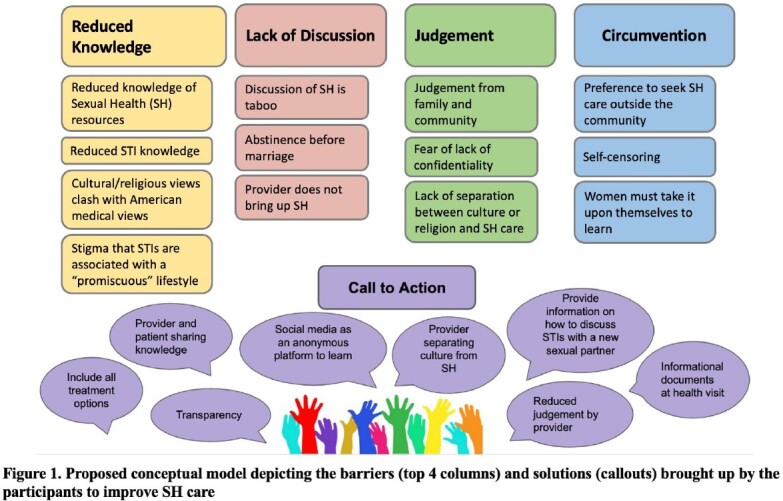

**Conclusion:**

We identified several themes underlying barriers to SH care for MENA immigrant women living in the US. We hope this project will fuel future research and policy efforts to raise awareness of SH in this vulnerable population. Appropriate and targeted interventions will ultimately help to prevent morbidity and mortality from diseases of SH in these women.

**Disclosures:**

**All Authors**: No reported disclosures

